# Author Correction: Estrogen receptor activation remodels *TEAD1* gene expression to alleviate hepatic steatosis

**DOI:** 10.1038/s44320-025-00126-0

**Published:** 2025-06-11

**Authors:** Christian Sommerauer, Carlos J Gallardo-Dodd, Christina Savva, Linnea Hases, Madeleine Birgersson, Rajitha Indukuri, Joanne X Shen, Pablo Carravilla, Keyi Geng, Jonas Nørskov Søndergaard, Clàudia Ferrer-Aumatell, Grégoire Mercier, Erdinc Sezgin, Marion Korach-André, Carl Petersson, Hannes Hagström, Volker M Lauschke, Amena Archer, Cecilia Williams, Claudia Kutter

**Affiliations:** 1 Department of Microbiology, Tumor, and Cell Biology, Karolinska Institute, Science for Life Laboratory, Solna, Sweden; 2 Department of Medicine, Integrated Cardio Metabolic Center, Karolinska Institute, Huddinge, Sweden; 3 Department of Protein Science, KTH Royal Institute of Technology, Science for Life Laboratory, Stockholm, Sweden; 4 Department of Biosciences and Nutrition, Karolinska Institute, Huddinge, Sweden; 5 Department of Physiology and Pharmacology, Karolinska Institute, Solna, Sweden; 6 Department of Women’s and Children’s Health, Karolinska Institute, Science for Life Laboratory, Solna, Sweden; 7 Department of Drug Metabolism and Pharmacokinetics, The Healthcare Business of Merck KGaA, Darmstadt, Germany; 8 Department of Medicine Huddinge, Karolinska Institute, Huddinge, Sweden; 9 Division of Hepatology, Department of Upper GI Diseases, Karolinska University Hospital Huddinge, Huddinge, Sweden; 10 Dr. Margarete Fischer-Bosch Institute of Clinical Pharmacology, Stuttgart, Germany; 11 University of Tübingen, Tübingen, Germany

## Abstract

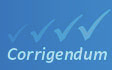

**Correction to:** *Mol Syst Biol* (2024) 20:374–402. 10.1038/s44320-024-00024-x | Published online 8 March 2024

The authors contacted the journal after they identified three errors in the Methods section.

**The Methods section is corrected**.

In the “Cell culture” section, there is a unit error.

The error is corrected from:

***Cell culture****.* HepG2 and AML12 cell lines were obtained from the American Type Culture Collection with certified genotype and were regularly tested for mycoplasma (Eurofins Genomics). HepG2 cells were cultured in Dulbecco’s Modified Eagle Medium (DMEM) supplemented with 10% fetal bovine serum (FBS, Hyclone, GE Healthcare) and 1% Penicillin-Streptomycin (PS, Sigma-Aldrich) while AML12 cells in DMEM/F-12 (Gibco) supplemented with 10% FBS, 1% PS, 1% Insulin-Transferrin-Selenium Sodium Pyruvate (Gibco) and 20 ng/mL Dexamethasone (Sigma-Aldrich) in T75 flasks at 37 °C and 5% CO_2_ atmosphere. Cells were passaged at a 1:6 ratio twice (HepG2) and three (AML12) times a week by aspirating the medium, gently washing the cells with PBS without Mg^2+^ (Sigma-Aldrich) and then detached using 2 mL of trypsin-EDTA solution (Sigma-Aldrich) for 3–5 min. Trypsin was inactivated with 8–10 mL of culture medium before passaging to a new flask.

To: (changes in bold):

***Cell culture****.* HepG2 and AML12 cell lines were obtained from the American Type Culture Collection with certified genotype and were regularly tested for mycoplasma (Eurofins Genomics). HepG2 cells were cultured in Dulbecco’s Modified Eagle Medium (DMEM) supplemented with 10% fetal bovine serum (FBS, Hyclone, GE Healthcare) and 1% Penicillin-Streptomycin (PS, Sigma-Aldrich) while AML12 cells in DMEM/F-12 (Gibco) supplemented with 10% FBS, 1% PS, 1% Insulin-Transferrin-Selenium Sodium Pyruvate (Gibco) and **40** **ng/mL Dexamethasone** (Sigma-Aldrich) in T75 flasks at 37 °C and 5% CO_2_ atmosphere. Cells were passaged at a 1:6 ratio twice (HepG2) and three (AML12) times a week by aspirating the medium, gently washing the cells with PBS without Mg^2+^ (Sigma-Aldrich) and then detached using 2 mL of trypsin-EDTA solution (Sigma-Aldrich) for 3–5 min. Trypsin was inactivated with 8–10 mL of culture medium before passaging to a new flask.

In the “siRNA-mediated TEAD1/Tead1 knockdown” section, there is a formatting error:

The error is corrected from:

***siRNA-mediated TEAD1/Tead1 knockdown****.* Confluent HepG2 (60–70%) or AML12 (80–90%) cells were trypsinized and electroporated with siRNAs targeting either TEAD1/Tead1 (SMARTpool) or a control non-targeting siRNA pool (ON-TARGETplusTM, Horizon Discovery). After washing cells once with OptiMEM (Invitrogen), 2 million cells were resuspended in 200 μl OptiMEM and incubated for 3 min with 2 μg (7.5 μL of a 20 μM stock) siRNA in a 4 mm cuvette (Bio-Rad) before being pulsed at 300V, 250 μF, in a Genepulser II (Bio-Rad). Immediately after electroporation, the cells were transferred to pre-heated (37 °C) phenol red-free DMEM (HepG2) or DMEM/F-12 (AML12) culture medium without antibiotics. Cells were collected at day four to determine knockdown efficiency and microscopy, and at day five for Seahorse analysis.

To (changes in bold):

***siRNA-mediated TEAD1/Tead1 knockdown****.* Confluent HepG2 (60–70%) or AML12 (80–90%) cells were trypsinized and electroporated with siRNAs targeting either TEAD1/Tead1 (SMARTpool) or a control non-targeting siRNA pool (**ON-TARGETplus**^**TM**^, Horizon Discovery). After washing cells once with OptiMEM (Invitrogen), 2 million cells were resuspended in 200 μl OptiMEM and incubated for 3 min with 2 μg (7.5 μL of a 20 μM stock) siRNA in a 4 mm cuvette (Bio-Rad) before being pulsed at 300V, 250 μF, in a Genepulser II (Bio-Rad). Immediately after electroporation, the cells were transferred to pre-heated (37 °C) phenol red-free DMEM (HepG2) or DMEM/F-12 (AML12) culture medium without antibiotics. Cells were collected at day four to determine knockdown efficiency and microscopy, and at day five for Seahorse analysis.

In the “RNA isolation and DNase treatment” section, there is a unit error.

The error is corrected from:

***RNA isolation and DNase treatment****.* Approximately 20 µg of flash-frozen liver tissue was homogenized in 700 µL QIAzol (QIAGEN) using a TissueLyzer II (QIAGEN, 2 min, 25 Hz, 2 times). The samples were incubated at room temperature for 5 min, before adding 140 µL chloroform (Sigma-Aldrich). This mixture was shaken for 15s, incubated for 3 min, and centrifuged at 9000 × g and 4 °C for 5 min. The aqueous phase was carefully removed, and an equal volume of isopropanol added. This mixture was incubated at room temperature for 10 min, before centrifugation at 20,000 × g and 4 °C for 10 min. Supernatant was removed and the pellet washed twice with 70% ethanol, air-dried, and resuspended in water. The isolated RNA was DNase treated using the Turbo DNase Kit (Thermo Fisher Scientific) according to the manufacturer’s instructions. In brief, 10 µg of RNA was treated with 2U DNase and 40U RNaseOUT (Thermo Fisher Scientific) at 37 °C for 30 min. DNase-treated RNA from mouse livers was incubated with DNase inactivation reagent (Turbo DNase kit) for 5 min under constant homogenization. The sample was centrifuged at 10,000 × g for 2 min to remove the inactivation reagent. To purify the obtained DNase-treated RNA, the RNA was diluted to 130 µL with water, before adding 20 µL sodium acetate (Thermo Fisher Scientific, 3M, pH 5.2), 1 µL GlycoBlue (Thermo Fisher Scientific) and 600 µL ice-cold 99.8% ethanol. Next, RNA was precipitated at −80 °C overnight, before centrifugation at 20,000 × g for 30 min, washing the pellet twice with 70% ethanol, air-drying and resuspending in water. DNase-treated RNA from liver spheroids was purified using an RNA clean and concentrator kit according to the manufacturer’s instructions (Zymo research). The RNA quality was assessed on a Bioanalyzer 2100 device using RNA Nano chips (Agilent Technologies) and only high quality RNAs (RIN > 6.5) were used for RNA-seq.

To (changes in bold):

***RNA isolation and DNase treatment****.* **Approximately 20** **mg of flash-frozen liver tissue** was homogenized in 700 µL QIAzol (QIAGEN) using a TissueLyzer II (QIAGEN, 2 min, 25 Hz, 2 times). The samples were incubated at room temperature for 5 min, before adding 140 µL chloroform (Sigma-Aldrich). This mixture was shaken for 15s, incubated for 3 min, and centrifuged at 9000 × *g* and 4 °C for 5 min. The aqueous phase was carefully removed, and an equal volume of isopropanol added. This mixture was incubated at room temperature for 10 min, before centrifugation at 20,000 × *g* and 4 °C for 10 min. Supernatant was removed and the pellet washed twice with 70% ethanol, air-dried, and resuspended in water. The isolated RNA was DNase treated using the Turbo DNase Kit (Thermo Fisher Scientific) according to the manufacturer’s instructions. In brief, 10 µg of RNA was treated with 2U DNase and 40U RNaseOUT (Thermo Fisher Scientific) at 37 °C for 30 min. DNase-treated RNA from mouse livers was incubated with DNase inactivation reagent (Turbo DNase kit) for 5 min under constant homogenization. The sample was centrifuged at 10,000 × *g* for 2 min to remove the inactivation reagent. To purify the obtained DNase-treated RNA, the RNA was diluted to 130 µL with water, before adding 20 µL sodium acetate (Thermo Fisher Scientific, 3M, pH 5.2), 1 µL GlycoBlue (Thermo Fisher Scientific) and 600 µL ice-cold 99.8% ethanol. Next, RNA was precipitated at −80 °C overnight, before centrifugation at 20,000 × *g* for 30 min, washing the pellet twice with 70% ethanol, air-drying and resuspending in water. DNase-treated RNA from liver spheroids was purified using an RNA clean and concentrator kit according to the manufacturer’s instructions (Zymo Research). The RNA quality was assessed on a Bioanalyzer 2100 device using RNA Nano chips (Agilent Technologies) and only high quality RNAs (RIN > 6.5) were used for RNA-seq.

Author statement:

These errors do not affect the conclusions of our study.

