# Supplementary material for: Estrogen receptor activation remodels TEAD1 gene expression to alleviate hepatic steatosis
Source: Mol Syst Biol. 2024 Mar 8;20(4):374–402. doi: 10.1038/s44320-024-00024-x (PMC10987545; doi:10.1038/s44320-024-00024-x)
Supplement: Supplementary file 1 — Appendix [file 44320_2024_24_MOESM1_ESM.pdf]

Appendix

Estrogen receptor activation remodels TEAD1 gene expression to alleviate hepatic steatosis

Sommerauer, Gallardo-Dodd, Savva, Hases, Birgersson, Indukuri, Shen, Carravilla, Geng, Søndergaard, Ferrer-Aumatell, Mercier, Sezgin, Korach-André, Petersson, Hagström, Lauschke, Archer, Williams, Kutter

Table of Contents

Appendix Figure S1 1
Appendix Figure S2 2
Appendix Figure S3 3

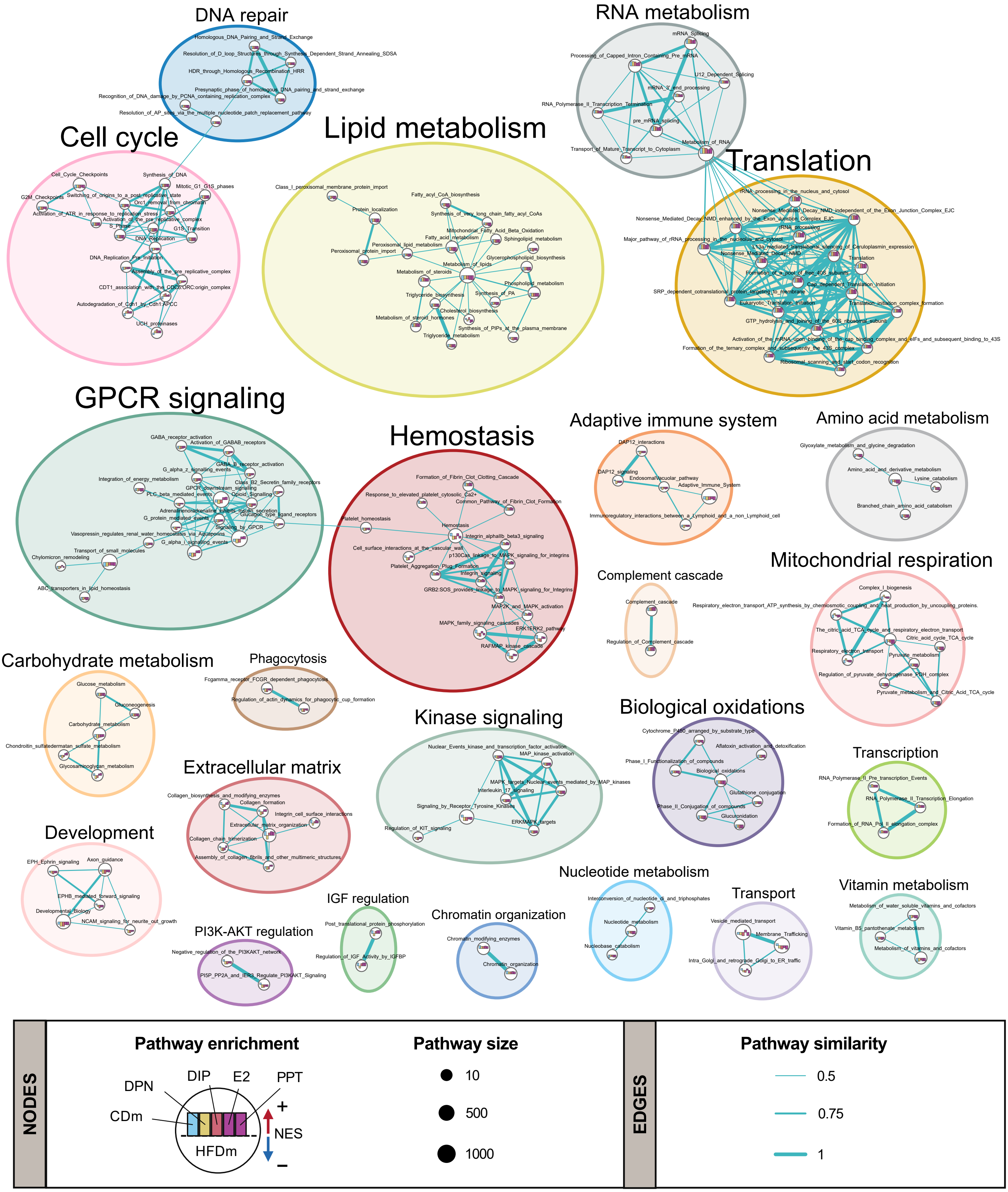

Appendix Fig S1. Cellular pathways are perturbed by ER-agonist treatments in an isoform-specific manner. The network displays pathway clusters (color-coded by large circles) and Reactome pathways (inner circles) identified by gene set enrichment analysis. Diameter of each inner circle indicates total number of genes per pathway (low: narrow, high: wide). Line thickness connecting Reactome pathways specifies term similarity (similarity > 0.5). Colored bars inside each inner circle display pathway enrichment of CD and ER-agonist-treated HFD relative to HFD males (normalized enrichment score, NES).

Node	Pathway cluster	# of genes	Non-reverted	Reverted	ERβ-specific	ERα-specific
1	Vitamin metabolism	128	3	5	4	6
2	Development	202	4	6	8	7
3	Hemostasis	351	10	13	10	12
4	Phagocytosis	67	0	2	1	2
5	Extracellular matrix	139	6	7	25	7
6	Carbohydrate metabolism	156	0	4	11	6
7	GPCR signaling	614	20	29	16	27
8	Lipid metabolism	487	24	37	9	19
9	Kinase signaling	308	2	6	9	11
10	PI3K/AKT regulation	72	1	2	2	3
11	Nucleotide metabolism	75	3	2	2	2
12	Mitochondrial respiration	109	0	0	1	3
13	Transcription	72	0	0	0	0
14	Cell cycle	302	3	4	5	6
15	Chromatin organization	137	0	1	1	1
16	Amino acid metabolism	152	6	3	0	8
17	IGF regulation	87	3	2	10	6
18	Transport	440	8	5	4	13
19	Translation	320	0	0	0	3
20	DNA repair	64	6	1	1	0
21	Biological oxidations	151	11	18	1	14
22	Complement cascade	40	2	3	4	1
23	RNA metabolism	487	1	0	0	4
24	Adaptive immune system	427	5	3	5	14

Axis (each bar column): 0 9 18

DEG fraction (%)

Appendix Fig S2. ERβ activation alters a high number of genes involved in extracellular matrix organization and IGF regulation. The table illustrates the number of expressed genes, deregulated genes for non-reverted (rose), reverted (denim), ERβ-specific (DPN-DIP, ochre) and ERα-specific (E2-PPT, violet) gene sets (Figure 2B) for each parent term. The bar length (x-axis) indicates the percentage of deregulated genes compared to the total number of genes per parent term.

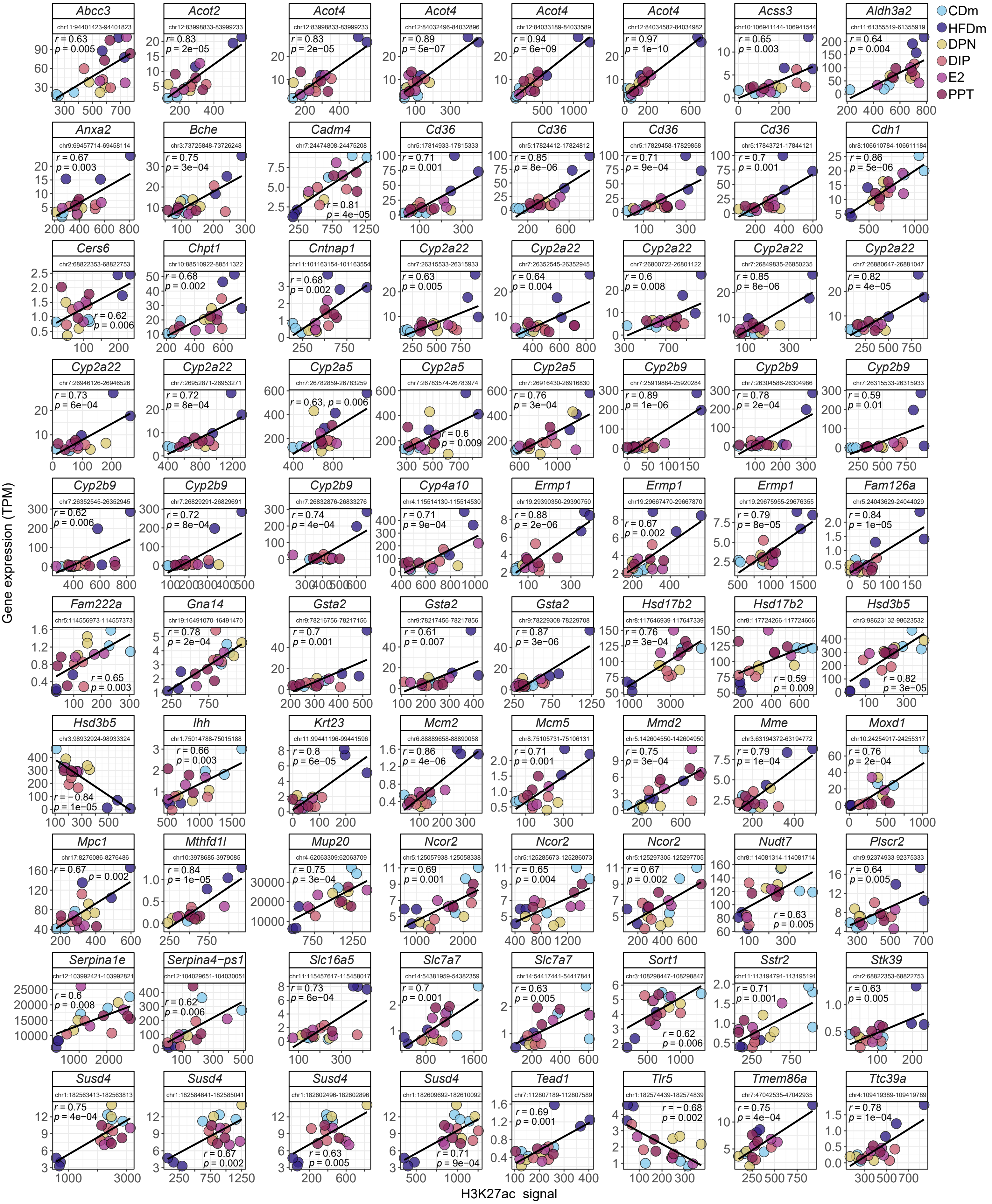

Appendix Fig S3. H3K27 acetylation - gene expression correlations identify 80 estrogen-sensitive enhancer-gene pairs. Scatter plots correlate expression (TPM, y-axis) of genes reverted by ER agonists and their respective paired estrogen-sensitive enhancers (H3K27ac signals, x-axis) in livers of male mice on different diets and ER-agonist treatments. All replicates are shown. Enhancer coordinates (400 bp window around the enhancer summit), Pearson correlation coefficient (r) and p-value (p) are indicated in each box.
