# Supplementary material for: Estrogen receptor activation remodels TEAD1 gene expression to alleviate hepatic steatosis
Source: Mol Syst Biol. 2024 Mar 8;20(4):374–402. doi: 10.1038/s44320-024-00024-x (PMC10987545; doi:10.1038/s44320-024-00024-x)
Supplement: Supplementary file 17 — Expanded View Figures [file 44320_2024_24_MOESM17_ESM.pdf]

Expanded View Figures

Figure EV1. Male but not female mice exhibit HFD-induced liver steatosis.

(**A–C**) Bar charts display physiological parameters assessed upon sacrifice in female (f) and male (m) mice (n = 4 mice per condition, +SD), which were previously examined (Hases et al, 2020). (**A**) Total weight, (**B**) liver weight and (**C**) blood glucose after 2 h fasting were measured. Color gradient indicates female and male mice on different diets (CD or HFD) and male mice upon ERα/β-agonist treatments (DPN, DIP, E2 and PPT). *P* values highlight non-significant, and asterisks indicate significant differences (**P* < 0.05, **<0.01, ****P* < 0.005, one-way ANOVA followed by Tukey's post-hoc test). (**D**) Hepatic cross-sections of four female and male mice on different diets (CD or HFD) and HFD males treated with an ER-agonist (DPN, DIP, E2 or PPT) were stained with hematoxylin and eosin. Images shown in Fig. 1B are highlighted in black boxes. Scale bar: 100 μm. (**E**) Two-way Venn diagram (left) shows the intersection and number (*n*) of genes deregulated exclusively in HFD male (top) or HFD female (bottom) mice compared to CD or deregulated in both sexes (middle). Bubble plots (right) show the top eight gene ontology terms (biological processes) for each intersected gene set. One-sided Fisher's exact test with Benjamini–Hochberg correction was used for the overrepresentation analysis.

© The Author(s)

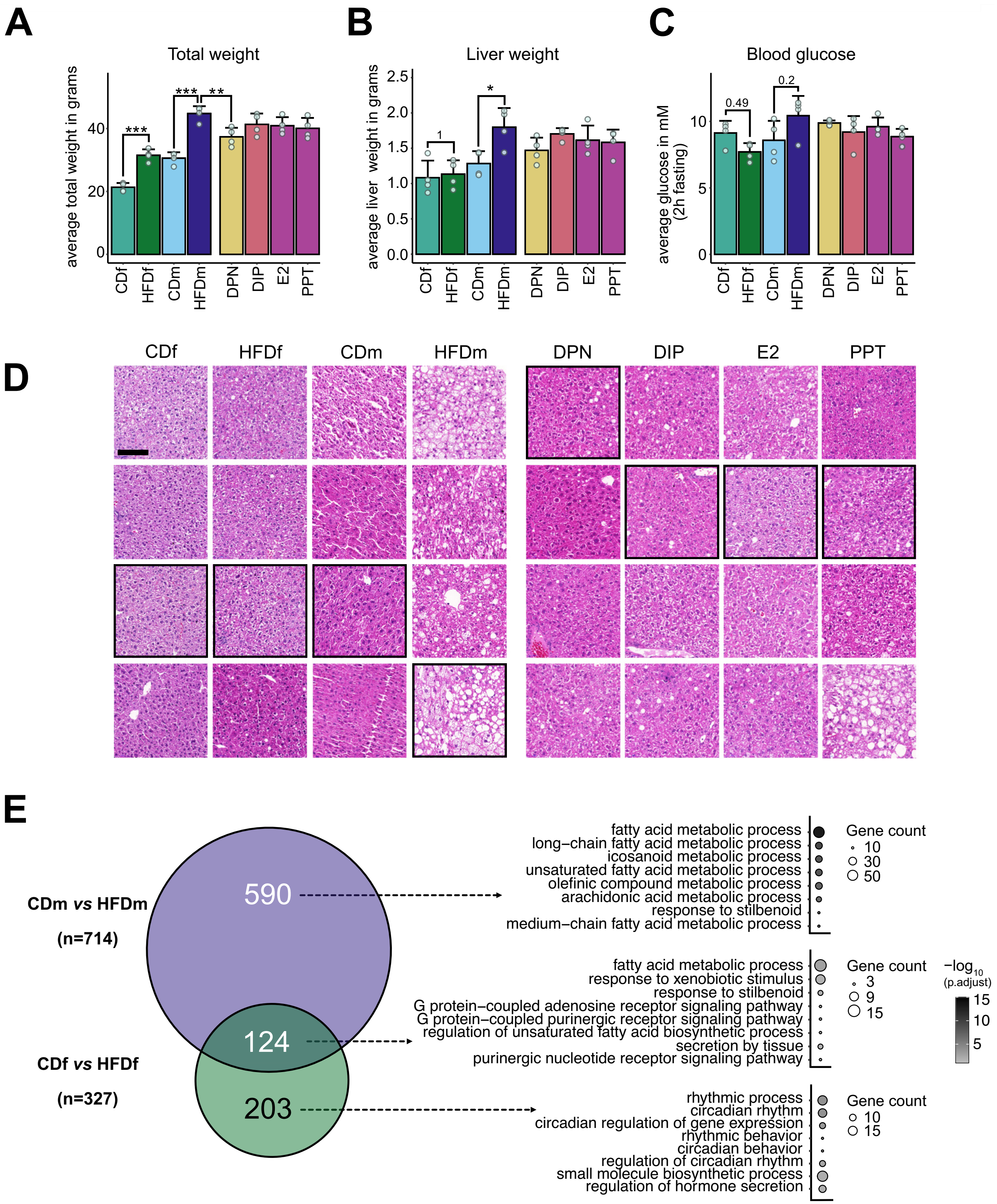
A
Total weight
average total weight in grams
***
***
**
CDf
HFDf
CDm
HFDm
DPN
DIP
E2
PPT
B
Liver weight
average liver weight in grams
1
*
C
Blood glucose
average glucose in mM (2h fasting)
0.49
0.2
D
CDf HFDf CDm HFDm DPN DIP E2 PPT
E
CDm vs HFDm
(n=714)
CDf vs HFDf
(n=327)
590
124
203
fatty acid metabolic process
long−chain fatty acid metabolic process
icosanoid metabolic process
unsaturated fatty acid metabolic process
olefinic compound metabolic process
arachidonic acid metabolic process
response to stilbenoid
medium−chain fatty acid metabolic process
Gene count
10
30
50
fatty acid metabolic process
response to xenobiotic stimulus
response to stilbenoid
G protein−coupled adenosine receptor signaling pathway
G protein−coupled purinergic receptor signaling pathway
regulation of unsaturated fatty acid biosynthetic process
secretion by tissue
purinergic nucleotide receptor signaling pathway
Gene count
3
9
15
−log10 (p.adjust)
15
10
5
rhythmic process
circadian rhythm
circadian regulation of gene expression
rhythmic behavior
circadian behavior
regulation of circadian rhythm
small molecule biosynthetic process
regulation of hormone secretion
Gene count
10
15

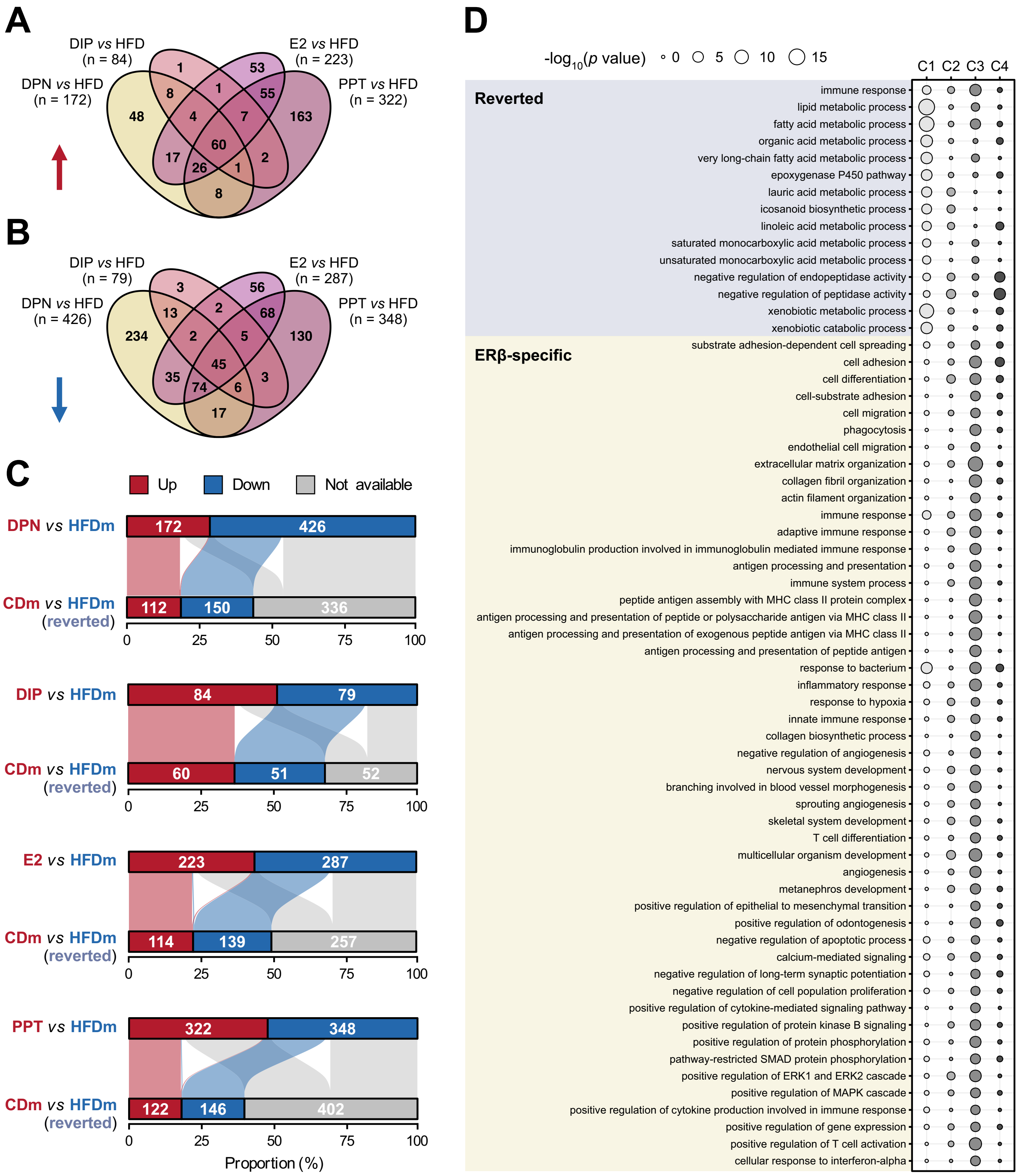
A
DIP vs HFD (n = 84)
E2 vs HFD (n = 223)
DPN vs HFD (n = 172)
PPT vs HFD (n = 322)
1
53
8
1
55
48
4
7
163
60
17
2
26
1
8
B
DIP vs HFD (n = 79)
E2 vs HFD (n = 287)
DPN vs HFD (n = 426)
PPT vs HFD (n = 348)
3
56
13
2
68
234
2
5
130
45
35
3
74
6
17
C
Up
Down
Not available
DPN vs HFDm
172
426
CDm vs HFDm (reverted)
112
150
336
DIP vs HFDm
84
79
CDm vs HFDm (reverted)
60
51
52
E2 vs HFDm
223
287
CDm vs HFDm (reverted)
114
139
257
PPT vs HFDm
322
348
CDm vs HFDm (reverted)
122
146
402
0
25
50
75
100
Proportion (%)
D
-log10(p value) 0 5 10 15
C1 C2 C3 C4
Reverted
immune response
lipid metabolic process
fatty acid metabolic process
organic acid metabolic process
very long-chain fatty acid metabolic process
epoxygenase P450 pathway
lauric acid metabolic process
icosanoid biosynthetic process
linoleic acid metabolic process
saturated monocarboxylic acid metabolic process
unsaturated monocarboxylic acid metabolic process
negative regulation of endopeptidase activity
negative regulation of peptidase activity
xenobiotic metabolic process
xenobiotic catabolic process
ERβ-specific
substrate adhesion-dependent cell spreading
cell adhesion
cell differentiation
cell-substrate adhesion
cell migration
phagocytosis
endothelial cell migration
extracellular matrix organization
collagen fibril organization
actin filament organization
immune response
adaptive immune response
immunoglobulin production involved in immunoglobulin mediated immune response
antigen processing and presentation
immune system process
peptide antigen assembly with MHC class II protein complex
antigen processing and presentation of peptide or polysaccharide antigen via MHC class II
antigen processing and presentation of exogenous peptide antigen via MHC class II
antigen processing and presentation of peptide antigen
response to bacterium
inflammatory response
response to hypoxia
innate immune response
collagen biosynthetic process
negative regulation of angiogenesis
nervous system development
branching involved in blood vessel morphogenesis
sprouting angiogenesis
skeletal system development
T cell differentiation
multicellular organism development
angiogenesis
metanephros development
positive regulation of epithelial to mesenchymal transition
positive regulation of odontogenesis
negative regulation of apoptotic process
calcium-mediated signaling
negative regulation of long-term synaptic potentiation
negative regulation of cell population proliferation
positive regulation of cytokine-mediated signaling pathway
positive regulation of protein kinase B signaling
positive regulation of protein phosphorylation
pathway-restricted SMAD protein phosphorylation
positive regulation of ERK1 and ERK2 cascade
positive regulation of MAPK cascade
positive regulation of cytokine production involved in immune response
positive regulation of gene expression
positive regulation of T cell activation
cellular response to interferon-alpha

© The Author(s)

Figure EV2. ERα/β-agonist treatments control molecular responses.

(**A**, **B**) Four-way Venn diagrams show intersections and numbers (*n*) of gene sets that are either (**A**) upregulated (red arrow) or (**B**) downregulated (blue arrow) by ER-agonist treatments compared to HFD in male mice ($n = 4$ mice per condition). (**C**) Horizontal bar plots (top) display the proportional frequency of up- (red) and downregulated (blue) genes in male mice on HFD compared to HFD male mice treated with an ERα/β-agonist (DPN, DIP, E2 or PPT). Horizontal bar plots (bottom) show the respective occurrences of the deregulated genes upon ER-agonist treatment in a diet comparison of male CD *versus* HFD. Genes significantly higher in CD male (red), higher in HFD male (blue) or unaltered (gray) are shown. Alluvial line width connecting upper and lower side indicate how many HFD-deregulated genes are recovered upon treatment (from red to red or blue to blue). Genes indicated in light gray are unchanged by HFD. Genes changed in both comparisons but not recovered upon treatment (from red to blue or blue to red) were categorized as non-reverted. (**D**) Bubble plot displays significantly enriched gene ontology terms (biological processes) per cluster (Fig. 2A, B) in reverted (denim) and ERβ-specific (ocher) gene sets. Circle color indicates the corresponding cluster, and circle size represent statistical significance of the gene ontology term (-$\log_{10}$ *P* value, low: narrow, high: wide). Hypergeometric test with Benjamini–Hochberg correction was used for the overrepresentation analysis.

© The Author(s)

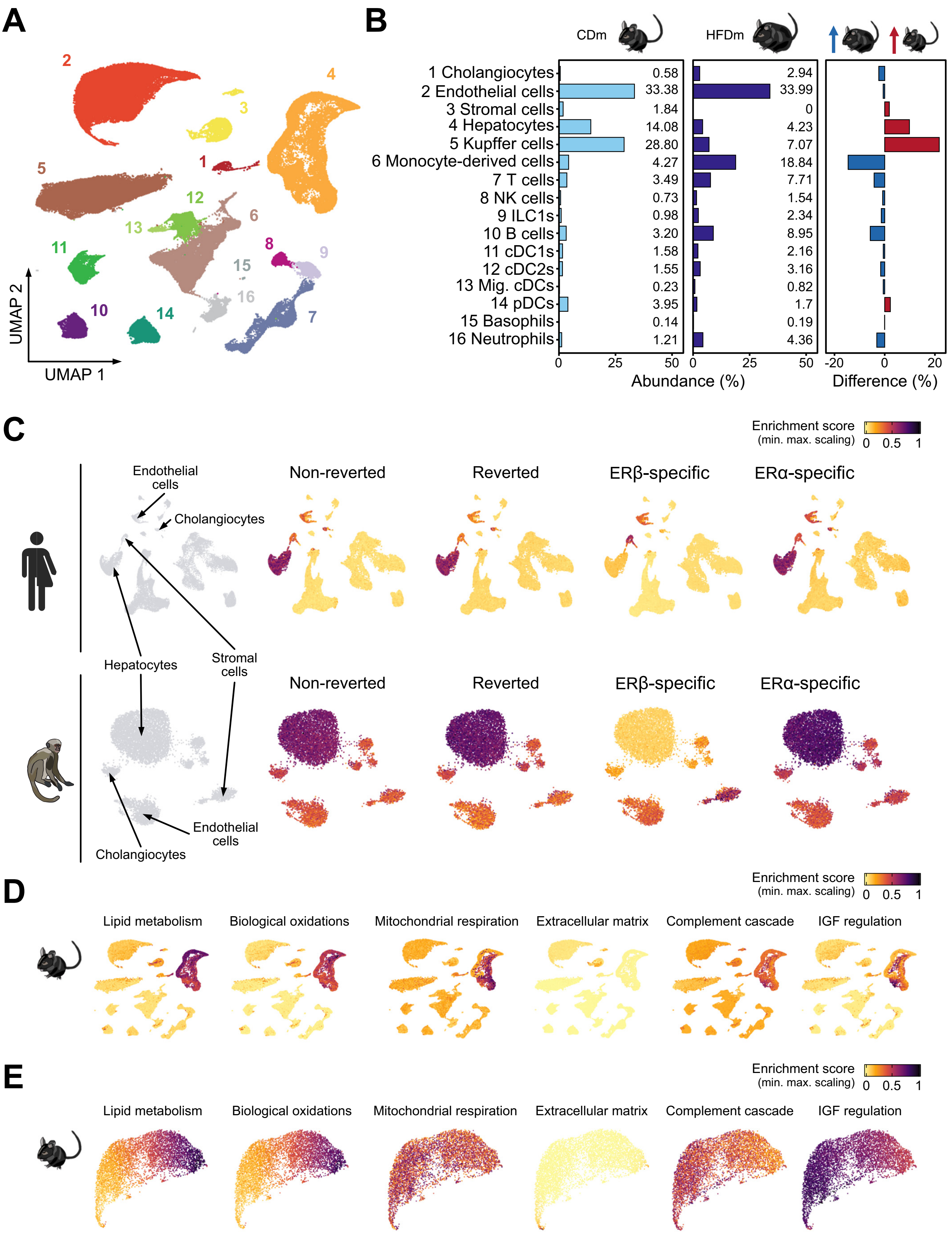
A
B
C
D
E
UMAP 1
UMAP 2
CDm
HFDm
1 Cholangiocytes
2 Endothelial cells
3 Stromal cells
4 Hepatocytes
5 Kupffer cells
6 Monocyte-derived cells
7 T cells
8 NK cells
9 ILC1s
10 B cells
11 cDC1s
12 cDC2s
13 Mig. cDCs
14 pDCs
15 Basophils
16 Neutrophils
0.58
33.38
1.84
14.08
28.80
4.27
3.49
0.73
0.98
3.20
1.58
1.55
0.23
3.95
0.14
1.21
2.94
33.99
0
4.23
7.07
18.84
7.71
1.54
2.34
8.95
2.16
3.16
0.82
1.7
0.19
4.36
Abundance (%)
Difference (%)
Enrichment score
(min. max. scaling)
Endothelial cells
Cholangiocytes
Hepatocytes
Stromal cells
Non-reverted
Reverted
ERβ-specific
ERα-specific
Lipid metabolism
Biological oxidations
Mitochondrial respiration
Extracellular matrix
Complement cascade
IGF regulation

 © The Author(s)

Figure EV3. ER-responsive molecular gene signatures show cell type-specificity and are maintained in primates.

(**A**) Single-cell map projected in UMAP space displays reference annotation of liver cell types (numbered according to Fig. EV3B). (**B**) Bar plots show the relative abundance in percent of distinct liver cell populations in control (left) and MASLD (middle) mice as well as their difference (right). Arrows indicate higher abundance in control (red) and MASLD (blue) mice. (**C**) Enrichment maps present liver cell type specificities of murine gene sets (defined in Fig. 2B) in human (top) and macaque (bottom) livers. Four relevant mouse- and primate-maintained cell types are labeled. (**D**) Enrichment maps show signal distribution of enriched pathways across liver cell types in mouse. (**E**) Spatial transcriptomics maps display liver zonation patters of the selected pathways. (**C–E**) Enrichment score: low: yellow, high: black.

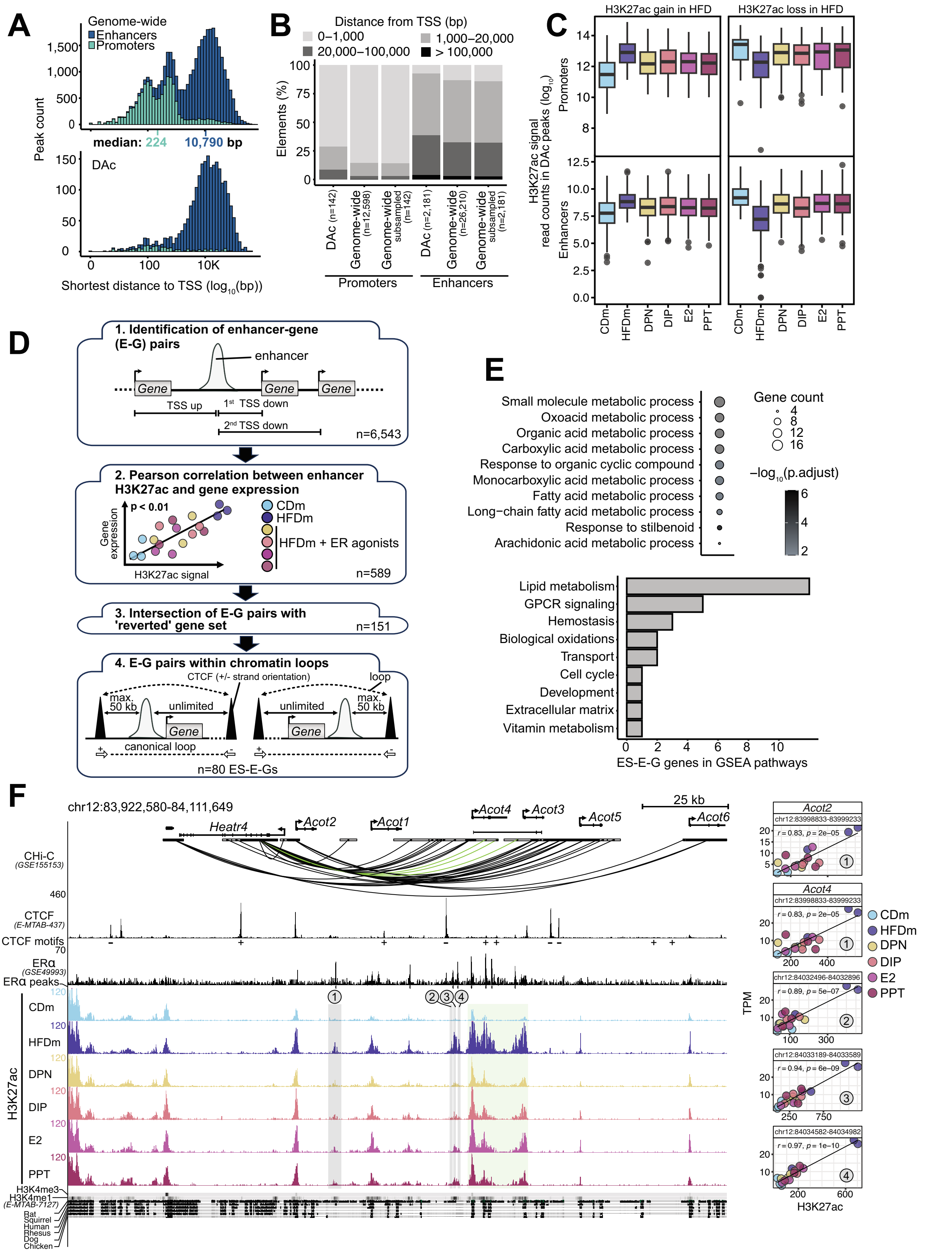
A
Genome-wide
Enhancers
Promoters
Peak count
median: 224 10,790 bp
DAc
Shortest distance to TSS (log10(bp))
B
Distance from TSS (bp)
0–1,000
1,000–20,000
20,000–100,000
> 100,000
Elements (%)
DAc (n=142)
Genome-wide (n=12,598)
Genome-wide subsampled (n=142)
DAc (n=2,181)
Genome-wide (n=26,210)
Genome-wide subsampled (n=2,181)
Promoters
Enhancers
C
H3K27ac gain in HFD
H3K27ac loss in HFD
H3K27ac signal read counts in DAc peaks (log10)
Promoters
Enhancers
CDm HFDm DPN DIP E2 PPT
D
1. Identification of enhancer-gene (E-G) pairs
enhancer
Gene
TSS up
1st TSS down
2nd TSS down
n=6,543
2. Pearson correlation between enhancer H3K27ac and gene expression
p < 0.01
Gene expression
H3K27ac signal
CDm
HFDm
HFDm + ER agonists
n=589
3. Intersection of E-G pairs with 'reverted' gene set
n=151
4. E-G pairs within chromatin loops
CTCF (+/- strand orientation)
loop
max. 50 kb
unlimited
canonical loop
n=80 ES-E-Gs
E
Small molecule metabolic process
Oxoacid metabolic process
Organic acid metabolic process
Carboxylic acid metabolic process
Response to organic cyclic compound
Monocarboxylic acid metabolic process
Fatty acid metabolic process
Long−chain fatty acid metabolic process
Response to stilbenoid
Arachidonic acid metabolic process
Gene count
−log10(p.adjust)
Lipid metabolism
GPCR signaling
Hemostasis
Biological oxidations
Transport
Cell cycle
Development
Extracellular matrix
Vitamin metabolism
ES-E-G genes in GSEA pathways
F
chr12:83,922,580-84,111,649
25 kb
Heatr4
Acot2
Acot1
Acot4
Acot3
Acot5
Acot6
CHi-C (GSE155153)
CTCF (E-MTAB-437)
CTCF motifs
ERα (GSE49993)
ERα peaks
H3K27ac
CDm
HFDm
DPN
DIP
E2
PPT
H3K4me3
H3K4me1 (E-MTAB-7127)
Rat
Squirrel
Human
Rhesus
Dog
Chicken
Acot2
chr12:83998833-83999233
r = 0.83, p = 2e−05
Acot4
chr12:83998833-83999233
r = 0.83, p = 2e−05
chr12:84032496-84032896
r = 0.89, p = 5e−07
chr12:84033189-84033589
r = 0.94, p = 6e−09
chr12:84034582-84034982
r = 0.97, p = 1e−10
TPM
H3K27ac
CDm
HFDm
DPN
DIP
E2
PPT

 © The Author(s)

Figure EV4. ERα/β-agonist treatments alter metabolic gene regulation.

(**A**) Histograms show frequency of genome-wide (top) or differentially acetylated (DAc, bottom) promoters (teal) and enhancers (dark blue) (*y* axis) with respect of distance to the closest annotated transcription start site (TSS, $\log_{10}$ scale, *x* axis). Median distance from nearest annotated TSS to promoters (teal) and enhancers (dark blue) is highlighted. (**B**) Stacked bar plot indicates percentage of DAc or genome-wide (all or downscaled to the number of DAc) promoter and enhancer elements binned according to the distance to the closest annotated TSS in bp. The numbers (*n*) of DAc and all promoter and enhancer regions are specified in parenthesis. (**C**) Box plots illustrate number of normalized (1× genome coverage) reads in peaks ($\log_2$) for the same regions and diet comparisons as in Fig. 4A ($n = 3$ mice per condition). Each box represents interquartile range (IQR) divided by the median (horizontal line), and whiskers span a maximum of 1.5×IQR. Outliers (circles) are shown. (**D**) Workflow displays identification of estrogen-sensitive enhancer-gene pairs (ES-E-Gs). First, for each enhancer, which is DAc in CDm *versus* HFDm ($n = 2181$), the three closest TSS are determined. The directionality of nearby gene transcription is not considered, instead the distance to the TSS ($n = 6543$). Second, Pearson correlation (*r*) and significance between H3K27ac signal and gene expression is assessed for each E-G pair, within individual replicates, considering only correlations of $P < 0.01$. Colors indicate male mice on different diets (CD or HFD) and upon ERα/β-agonist treatments. Third, E-G pairs with genes identified as ER-agonist treatment-reverted at the transcript level (Fig. 2B) and fourth, those with a CTCF peak within 50 kb under consideration of strand orientation, are selected. Numbers (*n*) of E-G pairs are displayed. (**E**) Bubble plot (top) shows the top 12-enriched gene ontology terms for the 49 genes part of ES-E-Gs ($-\log_{10}$ adjusted *p* value, low: narrow, high: wide). One-sided Fisher's exact test with Benjamini–Hochberg correction was used for the overrepresentation analysis. Horizontal bar plot (bottom) illustrates the contributions of the 49 genes to the GSEA pathway clusters (Fig. 3A). (**F**) Genome browser view (left) shows genomic regions (mm10) around *Acot2* and *Acot4* gene loci. Black boxes represent exons and UTRs. Arrows indicate directionality of gene transcription. Scale bar shows length of genomic regions in kilobases (kb). Promoter-capture Hi-C (CHi-C) 3D connections are shown for the *Acot* gene loci as black and green arcs. Genomic regions are enriched for CTCF (black peaks) with CTCF motif orientations determined with FIMO (indicated by plus or minus symbols), ERα (black peaks) and significant ERα peaks (black insets), H3K27ac in livers of CDm, HFDm and ER-agonist-treated HFDm, H3K4me3 and H3K4me1 (horizonal gray bars, dark: high, light: low). One replicate per condition is shown. The *y* axis of each track specifies normalized read density in livers of male mice. Genomic location of enhancers (numbered from 1 to 4) paired with the *Acot* gene loci are highlighted (gray vertical boxes). An additional enhancer-rich region across the *Acot4* and *Acot3* gene loci is shown (green vertical box). The degree of genomic sequence conservation at base resolution across selected vertebrates is shown (conserved: black, not conserved: white). Scatter plots (right) correlate *Acot2* and *Acot4* gene expression (TPM, *y* axis) and their respective paired enhancers (H3K27ac signals, *x* axis) in livers of male mice on different diets and ER-agonist treatments. All replicates are shown. Enhancer coordinates (400 bp window around the enhancer summit), Pearson correlation coefficient (*r*) and significance (*p*) are indicated in each box.

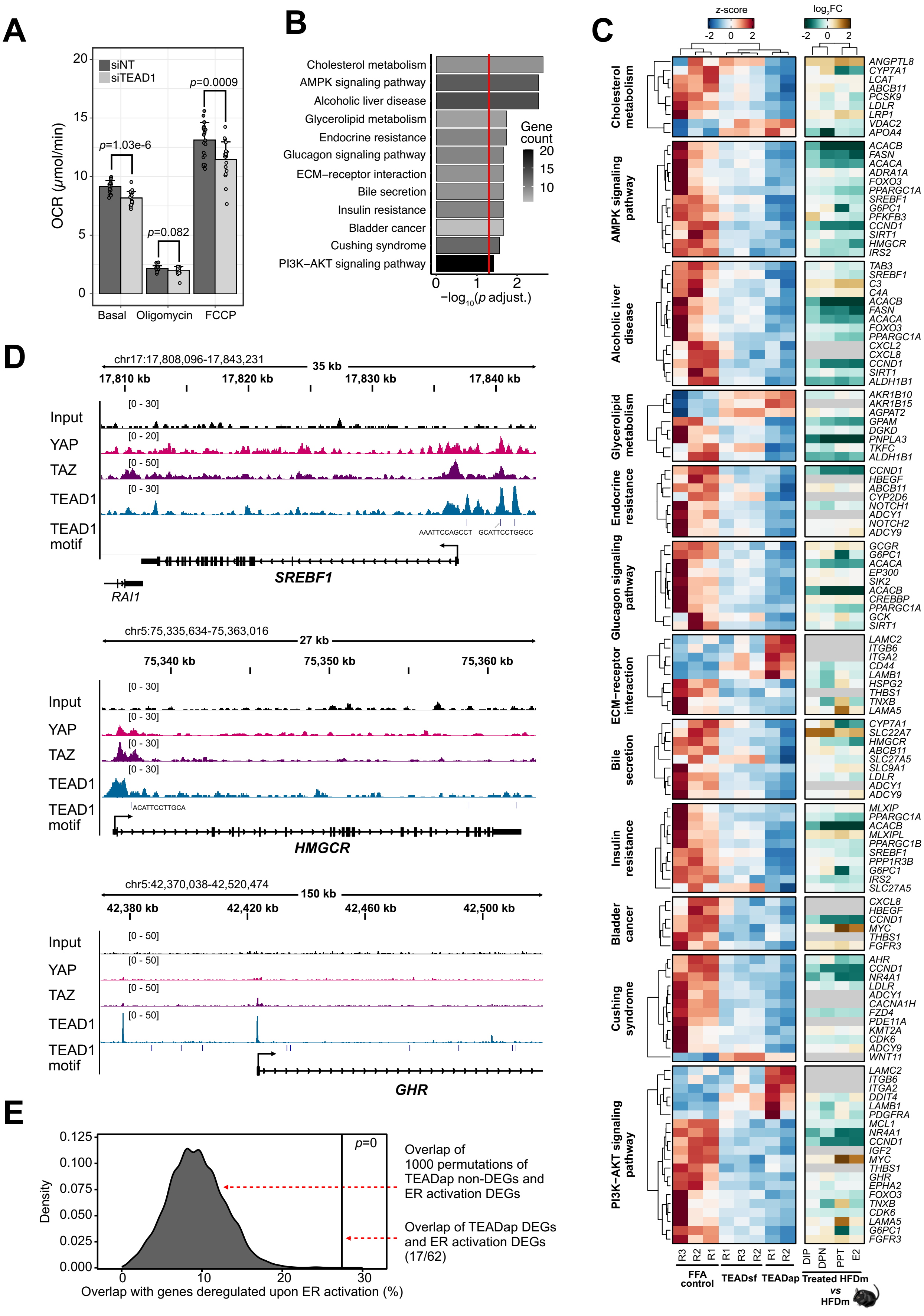

 © The Author(s)

Figure EV5. TEAD inhibition changes key cellular processes in the liver.

(**A**) Bar plot displays mean of basal, minimum, and maximum oxygen consumption rate in HepG2 cells with control (siNT, dark gray) and siRNA-mediated *TEAD1*-KD (siTEAD1, light gray) ($n = 63$ per condition, +SD). Dots indicate single wells across two biological replicates. Significant *P* values are indicated (two-sided *t* test). (**B**) Bar plot shows the adjusted *P* values (log_{10}) of the top 12 KEGG pathways enriched for genes deregulated upon TEADap treatment. Red vertical line indicates adjusted *P* value threshold ($P < 0.05$). Color gradient indicates gene number per pathway (low: gray, high: black). One-sided Fisher's exact test with Benjamini–Hochberg correction was used for the overrepresentation analysis. (**C**) Heatmap displays gene expression changes of the top 12 KEGG pathways altered upon TEADap treatment. Left panel indicates normalized gene expression (*z*-score) in free fatty acid-fed controls, TEADsf and TEADap treatments (low: blue, high: red). Right panel shows log_2FC for ER-agonist treatments compared to untreated male mice on HFD (low: green, high: brown). Gene names follow human nomenclature. Ortholog absence in mouse is specified (gray). (**D**) Genome browser views (IGV, hg38) show genomic regions around *SREBF1, HMGCR* and *GHR* gene loci. Genomic locations and sizes are indicated. The *y* axis of each track specifies normalized ChIP-seq read density (parenthesis) of YAP (pink), TAZ (purple) or TEAD1 (blue) in HUVEC cells (GSE163458). Vertical lines indicate TEAD1-binding sites identified by motif search. Blue boxes represent exons and UTRs, connecting lines indicate intronic sequences. Arrows indicate directionality of gene transcription. (**E**) Density plot shows the distribution of overlaps between 1000 random sets of genes unchanged by TEADap treatment and genes changed by ER-agonist treatments falling within the top 12-enriched KEGG pathways (gray peak). The percentage of overlapping genes changed in both TEADap and ER-agonist treatments for the top 12-enriched KEGG pathways is displayed (black vertical line). *P* value is indicated (permutation tests).

© The Author(s)
